# RNAi-Mediated Silencing of Catalase Gene Promotes Apoptosis and Impairs Proliferation of Bovine Granulosa Cells under Heat Stress

**DOI:** 10.3390/ani10061060

**Published:** 2020-06-19

**Authors:** Adnan Khan, Muhammad Zahoor Khan, Jinhuan Dou, Saqib Umer, Huitao Xu, Abdul Sammad, Hua-Bin Zhu, Yachun Wang

**Affiliations:** 1 Key Laboratory of Animal Genetics, Breeding and Reproduction, MARA, National Engineering Laboratory for Animal Breeding, College of Animal Science and Technology, China Agricultural University, Beijing 100193, China; dr.adnan93@cau.edu.cn (A.K.); zahoorkhattak91@yahoo.com (M.Z.K.); doujinhuan@cau.edu.cn (J.D.); drabdulsammad1742@yahoo.com (A.S.); 2 Embryo Biotechnology and Reproduction Laboratory, Institute of Animal Sciences, Chinese Academy of Agricultural Sciences, Beijing 100193, China; saqibumar33@hotmail.com (S.U.); xuhuitao104@163.com (H.X.); zhuhuabin@caas.cn (H.-B.Z.)

**Keywords:** bovine granulosa cells, catalase silencing, heat stress, oxidative stress, apoptosis, cell proliferation

## Abstract

**Simple Summary:**

Reduced fertility of modern-day dairy cattle across the world is implicated by the global warming phenomenon. Heat stress (HS) is well-known for compromising the normal physiological functions of granulosa cells (GCs) by promoting reactive oxygen species (ROS), which subsequently induce apoptosis, impair the biosynthesis of estrogen and progesterone, and disrupt the mitochondrial membrane potential. The catalase (CAT) enzyme serves a key antioxidant role in catalyzing the HS-induced ROS, thereby preventing the adverse effects of oxidative stress on cells. Therefore, the regulation of the CAT gene under HS has been the subject of increasing interest among researchers. However, no researches till date performed a functional validation of the CAT gene in bovine GCs under HS. For instance, we silenced the CAT gene using *si*RNA in GCs and found that the silencing of CAT aggravated the HS-induced damages to these cells, depicting a protective role of the CAT gene under HS. Thus, the regulation of CAT under HS could be used as an effective molecular marker to enhance the fertility in heat-stressed dairy cows.

**Abstract:**

Heat stress in dairy cattle is recognized to compromise fertility by altering the functions of ovarian follicle-enclosed cells, e.g., oocyte and granulosa cells (GCs). Catalase is an antioxidant enzyme that plays a significant role in cellular protection against oxidative damage by the degradation of hydrogen peroxide to oxygen and water. In this study, the role and mechanism of CAT on the heat stress (HS)-induced apoptosis and altered proliferation of bovine GCs were studied. The catalase gene was knocked-down successfully in bovine GCs at both the transcriptional and translational levels. After a successful knockdown using *si*RNA, GCs were divided into HS (40 °C + NC and 40 °C + CAT *si*RNA) and 38 °C + NC (NC) groups. The GCs were then examined for ROS, viability, mitochondrial membrane potential (MMP), cell cycle, and biosynthesis of progesterone (P4) and estrogen (E2) hormones. The results indicated that CAT silencing promoted ROS production and apoptosis by up-regulating the Bcl-2-associated X protein (BAX) and Caspase-3 genes both at the transcriptional and translational levels. Furthermore, the knockdown of CAT markedly disrupted the MMP, impaired the production of P4 and E2, altered the progression of the G1 phase of the cell cycle, and decreased the number of cells in the S phase. This was further verified by the down-regulation of proliferating cell nuclear antigen (PCNA), CyclinB1, steroidogenic acute regulatory protein (STAR), and cytochrome P450 family 11 subfamily A member 1 (Cyp11A1) genes. Our study presented a novel strategy to characterize how CAT can regulate cell proliferation and apoptosis in GCs under HS. We concluded that CAT is a broad regulatory marker in GCs by regulating apoptosis, cellular progression, and simultaneously by vital fluctuations in hormonal signaling. Our findings infer a crucial evidence of how to boost the fertility of heat-stressed cows.

## 1. Introduction

Heat stress (HS) is one of the abiotic stressors that influence the ovarian function [1], and subsequently reduces the maturation and fertilization ability of oocytes [2]. The marked decline in fertility during the periods when ambient temperatures are highest can approach 50% compared with the other seasons of the year. Moreover, the recovery of fertility during the autumn months is significantly delayed after the period when the environmental temperatures are highest during the summer months, showing that HS may have long-lasting effects on reproductive processes [3,4,5]. There is considerable evidence that HS impairs folliculogenesis, granulosa cell (GC) functions, and oocyte maturation, comprising reduced hormone synthesis, increased oxidative stress and apoptosis, and disrupted mitochondrial function [6,7,8,9,10,11,12].

Follicular GCs play a key role folliculogenesis and are the target of follicular growth-based researches. During folliculogenesis, the proliferation of GCs and hormone biosynthesis offer a crucial environment needed for follicular development [13]. Follicular growth and oocyte maturation are dependent on the proliferation and differentiation of GCs [14,15]. Increasing evidence suggests that HS promotes ROS production, such as singlet oxygen (O_2_), hydrogen peroxide (H_2_O_2_), superoxide radical (O2-), and hydroxyl radicals (OH), thus leading to oxidative damage by boosting up lipid peroxidation, protein oxidation and degradation, and DNA breakage [16,17,18,19,20]. Moreover, this oxidative stress results in apoptosis and reduced proliferation of GCs [21,22]. Additionally, ROS can consequently impair the competence of bovine oocytes to be matured and fertilized into quality embryos in vitro [23]. To counteract oxidative stress, a coordinated action between enzymatic, i.e., (CAT, superoxide dismutase (SOD), GPX, glutathione-S-transferases (GSTs), and glutathione reductase (GR)) and non-enzymatic (glutathione, ascorbate, and tocopherols) antioxidants plays a very significant role [24,25,26].

Among them, CAT is known to play a crucial role in antioxidant defense pathways by effectively catalyzing, in peroxisomes and glyoxysomes, the conversion of two molecules of H_2_O_2_ to two molecules of H_2_O and an O_2_ (2H_2_O_2_ → 2H_2_O + O_2_), thus neutralizing the H_2_O_2_ toxicity [27,28]. Catalase, a tetrameric protein, comprises four heme groups that react with H_2_O_2_ [29]. Due to its highest catalytic efficacies, CAT has been considered as a major antioxidant enzyme in reproductive processes. A single CAT molecule can convert millions of H_2_O_2_ molecules into H_2_O and O_2_ per second (sec) [30]. Subsequently, this process avoids the transformation of H_2_O_2_ into OH and other noxious radicals [31]. Previously, CAT regulation has been studied at both the enzymatic and transcriptional levels in human spermatozoa, seminal plasma of fertile and infertile males [32,33], maize [31], cucumber [34] olive [35], broccoli [36], and banana [37]. Nonetheless, to our knowledge, no prior studies have validated its function in bovine GCs.

Thus, we hypothesized that the silencing of CAT could regulate apoptosis and cell proliferation in bovine GCs under HS. Currently, RNA interference (RNAi) has shown its efficacy in the modulation and variation of a specific gene knockdown in many species [38]. Hence, the current study was aimed to explore the role of CAT in the intracellular ROS accumulation, cell viability, MMP, cell cycle control, and apoptosis of GCs, which was further proved by the expressions of associated genes. Here, we show that CAT played a critical role in the regulation of apoptosis and the progression of the GC cycle under HS. Furthermore, we also demonstrate that CAT has a role in the regulation of steroidal hormones (E2 and P4) biosynthesis. Importantly, we show that the silencing of CAT induced intracellular accumulation of ROS that results in GCs apoptosis and cell cycle arrest. Likewise, MMP and biosynthesis of steroidal hormones in GCs were also compromised, depicting that CAT avoids apoptosis by affecting the biosynthesis of intracellular hormone regulation. Together, our data provide a better understanding of CAT local regulation within GCs and overall, in reproductive events.

## 2. Materials and Methods

### 2.1. Ethical Approval

The research procedures and protocols for bovine ovaries collection were evaluated and permitted by the institutional animal care and use committee at China Agricultural University Beijing, China under the permission number: DK996.

### 2.2. Isolation, Culture and Treatment of GCs

Ovaries acquired from Holstein cattle from a local abattoir were transported to the lab in saline supplemented with 100 U/mL penicillin and 0.1 mg/mL streptomycin, within 2 h (hours). Amber-colored follicular fluid was collected from small and healthy follicles (2–6 mm) using 20 mL sterile syringe (B-Braun, Germany) and was filtered with a 40 μm filter before transferring to 15 mL conical tubes (Corning, NY, USA). The filtrate with GCs was centrifuged at 1500× *g* for 5 min (min). After centrifugation, the filtrate was washed with warm phosphate buffer saline (PBS) and resuspended in culture medium (DMEM/F-12, Gibco, Life Technologies Inc., Grand Island, NY, USA) added with 10% fetal bovine serum (FBS, Gibco, Life Technologies Inc., Grand Island, NY, USA). Cells (6 × 10^6^ cells per well) were then pre-cultured in a 6-well plate containing 2 mL of DMEM/F-12 enriched with 10% FBS and incubated at 38 °C under 5% CO_2_ in humidified air. After 48 h of pre-culture, non-adherent cells were removed by changing the fresh medium. The remaining cells were with the confluence level of more than 80% and a triangular or polygonal shape containing larger nuclei and expressed follicle-stimulating hormone receptor (FSHR), specific for GCs. The GCs were subjected to different treatment conditions. In order to establish a HS model, three parallel groups were set up, namely 38 °C + NC (NC), 40 °C + NC, and 40 °C + small interfering CAT (CAT *si*RNA; that knocks down the CAT gene). Cells were further cultured for 24 h at 38 °C. Soon after the culture, cells and culture media were collected for further experiments.

### 2.3. Identification of GCs by Immunofluorescence

Bovine GCs were seeded on coverslips in a 12-well plate for 24 h. The cells attached with the bottom were fixed with absolute cold methanol for 20 min, and permeabilized with 200 μL 0.1% Triton X-100 (Sigma-Aldrich, St. Locus, MI, USA) in PBS for 5 min. After washing with PBS, cells were loaded with 5% goat serum for 1 h and incubated with anti-FSHR antibody (1:500, Sigma-Aldrich, St. Locus, MI, USA) at 4 °C overnight. Moreover, cells were exposed to FITC-conjugated secondary antibody (1:1000, Sigma-Aldrich, St. Locus, MI, USA) for 2 h in the dark. Cells were then washed two times with PBS and stained with PI stain (Sigma-Aldrich, St. Locus, MI, USA) for staining nuclear DNA. Cells were mounted and observed under a fluorescence microscope (Olympus, Tokyo, Japan).

### 2.4. Immunohistochemistry

Following collection, ovaries were washed with a warm 0.9% normal saline and rinsed in pre-warm 70% ethanol for 30 s, followed by washing with warm PBS three times. Ovarian follicles with a diameter of 2–6 mm were isolated using a sterilized scalpel. After collection, follicles were washed three times with warm PBS. The follicles were then divided into two groups; control (38 °C) and 40 °C for 2 h under 5% CO_2_ in humidified air. Soon after treatment, ovarian follicles were aspirated using a 20 mL sterile syringe (B-Braun, Germany) and then embedded in optimal cutting temperature medium for sectioning. Sections (7 μm) were fixed with 4% paraformaldehyde and then permeabilized with 1% Triton X-100 for 10 min. After fixing, sections were washed with PBS two times and were incubated with antibodies against CAT (Sigma-Aldrich, St. Locus, MI, USA, dilution 1:500) for 2 h. Furthermore, the sections were washed again and incubated with secondary antibodies conjugated to horseradish peroxidase (HRP) for 1 h. Finally, the sections were developed by the addition of 3,3’-diaminobenzidine (DAB) substrate. To visualize of the positively stained cells in the follicular wall, a light contrast microscope was used. Likewise, a Nikon Eclipse, TE 2000-U fluorescence microscope was used for obtaining images.

### 2.5. Production of siRNA and Transfection

For the CAT knockdown, RNA interference was done using *si*RNA directed against cow CAT (*si*CAT) (the sense and antisense sequence of *si*RNA are 5′-GCGAAGGUGUUUGAGCAUATT-3′ and 5′-UAUGCUCAAACACCUUCGCTT-3′, respectively) and negative control (NC) purchased from Gene Pharma (Shanghai, China). Bovine GCs were seeded in 6-well plates and transfected with LipofectamineTM 3000 (Invitrogen, Carlsbad, CA, USA) following the instruction of the manufacturer. Afterwards, cells were resuspended in DMEM/F-12 culture medium and incubated at 38 °C under 5% CO_2_ in humidified air. GCs were collected for protein and RNA extraction after 24–48 h of transfection. Furthermore, culture media were collected for the hormonal estimation.

### 2.6. Total RNA Extraction and RT-qPCR

To extract the total RNA from the GCs, an RNA kit (Tiangen, Beijing, China) was used as instructed by the manufacturer. The concentration of RNA was estimated with a NanoDrop 2000 spectrophotometer (Thermo Scientific, Waltham, MA, USA). Gene expression was measured by real-time PCR analysis using iTaq™ Universal SYBR^®^ Green Supermix (Bio-Rad Laboratories GmbH, Feldkirchen, Germany) in applied Biosystem^®^ StepOnePlus™ (Applied biosystems, Foster City, CA, USA). The relative expression of each target gene (CAT, BAX, Caspase-3, PCNA, CyclinB1, STAR, and Cyp11A1) was normalized to that of GAPDH. Table 1 shows the list of gene-specific primers designed by primer blast (http://www.ncbi.nlm.nih.gov/tools/primer-blast/) and that were double-checked by Oligo v7 (Molecular Biology Insights, Inc., Cascade, CO, USA). The second derivative maximum method was applied for the data acquisition. The 2^−ΔΔCT^ method was used to calculate the gene expression level [39].

### 2.7. Western Blot

Bovine GCs from each group were lysed with RIPA lysis buffer (Beyotime, Shanghai, China) having proteinase inhibitors. BCA Protein Assay Kit (Beyotime) was used for the determination of the extracted protein concentration. Equal amounts of protein (10 μg) were subjected to 10% SDS-polyacrylamide gel electrophoresis, and transferred onto a polyvinylidene difluoride (PVDF) membrane for 60 min. For blocking the membrane, skim milk in Tris-buffered saline (20 mM Tris-HCl, pH 7.6, 137 mM NaCl) with 0.1% Tween-20 (TBST) was used for 60 min at 38 °C and incubated with primary antibodies: anti CAT, BAX, Caspase-3, PCNA, CyclinB1, STAR, Cyp11A1, and β-actin (Cell Signaling Technology, Beverly, MA, USA), at 4 °C overnight. After washing with TBST thrice, membranes were incubated with HRP-conjugated secondary antibody (Zhongshan Biotechnology, Beijing, China) for 1 h at room temperature. The bands of protein were visualized through a chemiluminescence detection kit (Tanon, Shanghai, China). The bands were measured using the Image J 1.44p software and β-actin was used as a reference protein for normalization.

### 2.8. Estimation of Intracellular ROS

Intracellular ROS were measured using the oxidation sensitive probe 6-carboxy-2′,7′-dichlorodihydrofluorescein diacetate (H_2_DCF-DA) (Invitrogen, Carlsbad, CA, USA), which is oxidized to fluorescent DCF by intracellular ROS. To examine the net intracellular production of ROS by using a fluorescence microscope (Olympus, Tokyo, Japan), GCs were trypsinized 48 h after culturing in the absence or presence of the different treatment conditions (NC, 40 °C + NC, and 40 °C + CAT *si*RNA). Following treatment, 10 μmol/L H_2_DCFDA was added to each well and incubated for 30 min at room temperature in the dark. After washing with 0.1% PVA/DPBS, the fluorescence of GCs was observed under a fluorescence microscope (Olympus, Tokyo, Japan).

### 2.9. Estimation of GCs Apoptosis

Bovine GCs were detected for apoptosis using the Annexin V-FITC kit (Beyotime Biotechnology, Shanghai, China) following the instructions of the manufacturer. After the indicated treatments, GCs were trypsinized, collected, and incubated with Annexin V-FITC for 20 min in the dark and PI for 2 min. Flow cytometry (BD Biosciences, San Jose, CA, USA) was done immediately afterwards. The apoptotic rate was expressed in percentage. The data were analyzed by the Flowjo software (version Win64-10.4.0)

### 2.10. Analysis of Cell Cycle

The cell cycle and apoptosis analysis kit (Beyotime, Shanghai, China) was used to determine the transition of the GCs’ cell cycle. GCs from each treated group were harvested in a 15 mL Falcon tube, followed by centrifugation at 1000× *g* for 5 min. A minimum of 1 × 10^6^ GCs was fixed in 70% chilled ethanol overnight. After removing ethanol, the GCs’ pellets were washed twice with 500 µL of 1× PBS. Moreover, cells were incubated with 50 µg/mL of PI and 50 µg/mL of RNase at 37 °C for 30 min in the dark and immediately processed in FACS Calibur (BD Biosciences, San Jose, CA, USA). The data obtained from the FL2-A channel using the ModFit LT Version 4.1 software (http://www.vsh.com/products/mflt/index.asp) were used to estimate the percentage of cells in each cell division phase (G0-G1, S, and G2-M).

### 2.11. Mitochondrial Membrane Potential Analysis

To evaluate the effect of all treatments on the MMP of GCs, the MMP assay kit with JC-1 (Beyotime, Shanghai, China) was used. GCs were enzymatically digested and harvested in 15 mL conical tubes. Afterwards, GCs were washed with warm PBS and stained with MMP assay following the manufacturer’s instructions. Flowcytometry using (FACS) a Calibur flow cytometer was done for counting the stained cells. The Flowjo software (version Win64-10.4.0) was used to analyze the data.

### 2.12. Estimation of Cell Viability

Bovine GCs from all treated groups were evaluated for cell viability using an MTT cell proliferation and cytotoxicity assay kit (njjcbio, Nanjing, China) following the instructions of the manufacturer. Primary GCs were seeded into 96-well plates. After the specified treatments (NC, 40 °C + NC, and 40 °C + CAT *si*RNA), each well was supplemented with 50 µL 1 × MTT solution and incubated for 4 h. After incubation, the mixture was substituted with 150 µL DMSO and absorbance was analyzed at 570 nm by an ELX microplate reader (BioTek, Winooski, VT, USA).

### 2.13. Determination of E2 and P4 Levels

Cell culture media from all groups (NC, 40 °C + NC, and 40 °C + CAT *si*RNA) were used for the estimation of the E2 and P4 levels. Their levels were estimated by using P4 and E2 ELISA kits (ENZO life sciences, Germany) following the instructions of the manufacturer.

### 2.14. Statistical Analysis

Data are presented as mean values ± SEM. Results were analyzed statistically using the SPSS 16.0 and GraphPad Prism5 software (GraphPad Software Inc, San Diego, CA, CA). The variations among all groups were analyzed using a one-way ANOVA followed by a multiple comparison post hoc test. Three biological replicates (*n* = 3) of GCs were used in each group. Differences at *p* < 0.05 were considered as statistically significant.

## 3. Results

### 3.1. Heat Stress Induced CAT Expression in Ovarian Follicle Tissues

**Figure 1:**
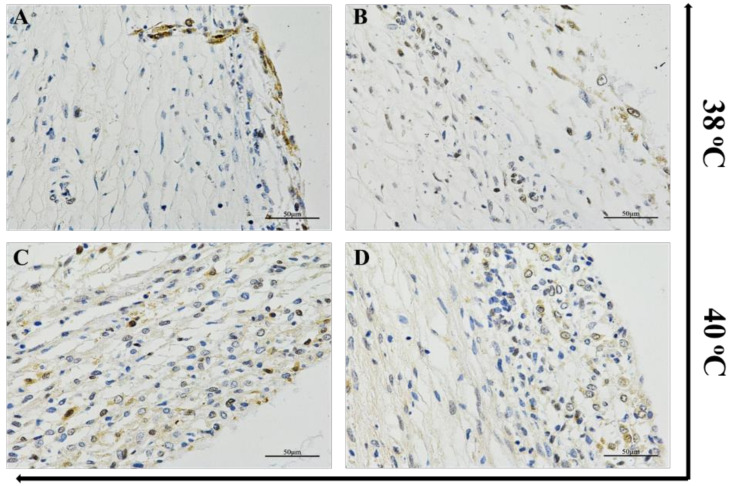
Detection of CAT expression in ovarian follicle. The appearance of bluish granules scattered within the follicular wall indicated an increased positive staining for the CAT protein in the heat-treated group (40 °C) (**C**,**D**) than the control (**A**,**B**), examined by immunohistochemistry. The stained sections were developed with DAB substrates and visualized under a light contrast microscope.

The expression of the CAT protein under HS was investigated using immunohistochemical staining. For instance, bovine ovarian follicles were divided into two groups: control (38 °C) and 40 °C for 2 h under 5% CO_2_ in humidified air, sectioned (7 μm), stained, and visualized under a light contrast microscope. The results showed that the appearance of bluish granules scattered within the follicular wall indicated an increased positive staining for the CAT protein in the heat-treated group as compared with the control (Figure 1).

### 3.2. Identification of GCs

**Figure 2:**
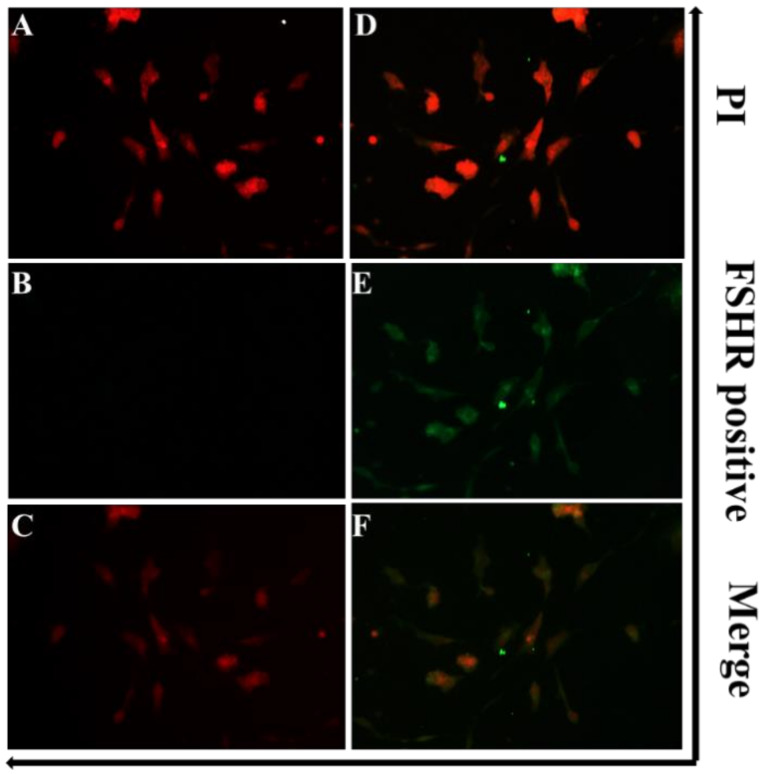
Identification of granulosa cells (GCs). PI was used for staining the cellular nuclei (**A**,**D**). The cultured cells were FSHR positive (**E**). (**B**) Negative control. (**C**) is the merged image of (**A**) and (**B**). (**F**) is the merged image of (**D**) and (**E**). Original magnification × 100.

Ovarian follicle contains a variety of cells (GCs, theca cells, and cumulus oophorus cells). GCs are the main focus of our study; therefore, immunofluorescence microscopy was done to isolate GCs from the rest of the follicular cells. GCs were either counterstained with PI or incubated with anti-FSHR antibody. Results showed that GCs were positive for FSHR (Figure 2).

### 3.3. Efficacy of CAT Transfection

**Figure 3:**
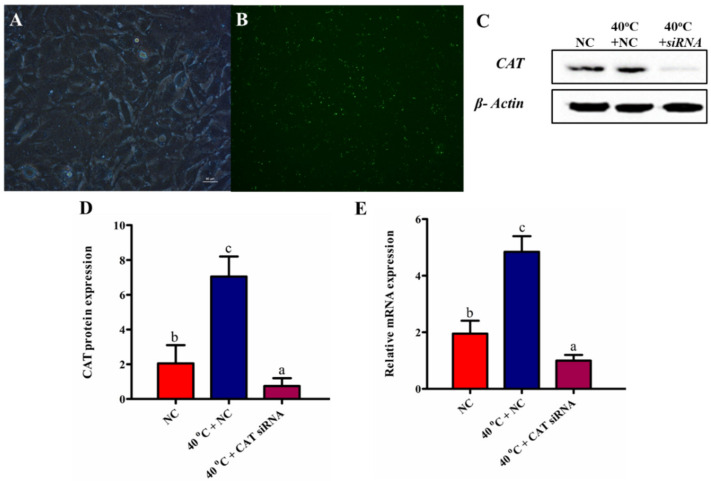
Detection of CAT *siRNA* efficacy in GCs (**A**,**B**). After transfection, the CAT protein expression was detected by Western blotting in GCs (**C**,**D**). Transcription levels of the CAT gene among all treated groups (**E**). Values are expressed as mean ± SEM of *n* = 3. Superscripts (a, b, c) represent a significant (*p* < 0.05) difference.

The silencing of CAT was done using small interfering RNAs directed against cow CAT (*si*CAT). The efficacy of *siCAT* in the cultured GCs (Figure 3A) was checked under a fluorescence microscope (Olympus, Tokyo, Japan) (Figure 3B). After successful transfection, the GCs were divided into different groups (NC, 40 °C + NC, and 40 °C + CAT *si*RNA). The expression of CAT was estimated among all groups both at the translational and transcriptional levels. Protein expression results revealed that the CAT gene was effectively silenced with *si*CAT (Figure 3C,D). Likewise, RT-qPCR showed that the CAT level was significantly (*p* < 0.05) reduced in GCs transfected with *si*CAT (Figure 3E).

### 3.4. Silencing of CAT Induced Intracellular ROS Accumulation under HS

**Figure 4:**
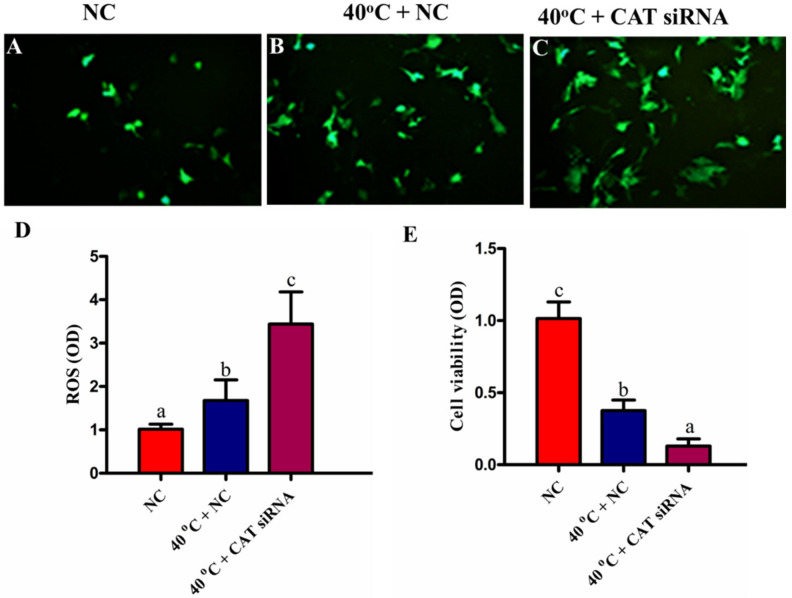
CAT regulates intracellular accumulation of ROS and viability of GCs under heat stress (HS). Intracellular ROS abundance measured through DCF fluorescence; NC (**A**) and HS (40 °C + NC and 40 °C + CAT *si*RNA) (**B**,**C**, respectively). Quantitative analysis of relative fluorescence emission (**D**). The cells transfected with CAT *si*RNA under HS were subjected to MMT cell viability assay (**E**). Values are expressed as mean ± SEM of *n* = 3. Superscripts (a, b, c) represent a significant (*p* < 0.05) difference.

To investigate the intracellular accumulation of ROS among all treated groups (NC, 40 °C + NC, and 40 °C + CAT *si*RNA), the oxidation sensitive probe H_2_DCF-DA (Invitrogen, Carlsbad, CA, USA) was used, which emits an increased fluorescence in response to intracellular generation of ROS. Our results revealed that the intracellular abundance of ROS was significantly (*p* < 0.05) higher in the 40 °C + NC group than the NC group. Likewise, the ROS generation in the 40 °C + CAT *si*RNA group was significantly (*p* < 0.05) more elevated than the 40 °C + NC group. These results showed that the CAT silencing further increased intracellular ROS production (Figure 4A–D). Therefore, our results showed that under HS, CAT is of crucial importance to regulate intracellular ROS production.

### 3.5. Silencing of CAT Altered Viability of GCs under HS

The viability of GCs was assessed by an MTT assay. We found that the viability of GCs was significantly (*p* < 0.05) lower in the 40 °C + NC group than that in the NC group. Likewise, a decrease in cell viability was noted in the 40 °C + CAT *si*RNA group than that in the NC and 40 °C + NC groups (Figure 4E). Our results depicted that the viability of GCs was compromised due to the oxidative stress induced by the ROS production under HS, and the silencing of CAT caused further damage, thus showing its important role in the regulation of cell viability.

### 3.6. Silencing of CAT Induced GCs Apoptosis under HS

**Figure 5:**
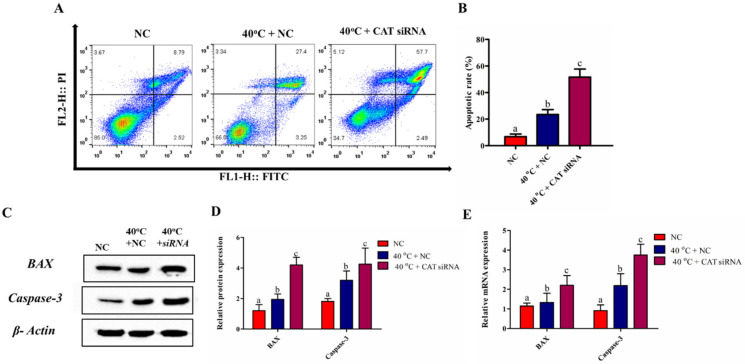
CAT regulates GCs apoptosis under HS. Apoptosis events were determined by FACS analysis after PI/annexin V staining in the GCs (**A**,**B**). The expression of BAX and Caspase-3 proteins were analyzed by Western blotting (**C**,**D**). β- Actin was used as a housekeeping gene. Similarly, the mRNA expression of BAX and Caspase-3 from GCs of the NC and treated groups (40 °C + NC and 40 °C + CAT *si*RNA) were evaluated by RT-qPCR. GAPDH was used as a reference gene (**E**). Values are expressed as mean ± SEM of *n* = 3. Superscripts (a, b, c) represent a significant (*p* < 0.05) difference.

An annexin V-FITC kit (Beyotime Biotechnology, China) was used to elucidate the role of CAT in the regulation of apoptosis in GCs under HS. The flow cytometry results showed that the apoptotic rate in the 40 °C + NC group was significantly (*p* < 0.05) higher than that in the NC group. Similarly, the quantitative results showed that the silencing of CAT in GCs at 40 °C significantly (*p* < 0.05) elevated the apoptotic rate when compared with both in the NC and 40 °C + NC groups (Figure 5A,B). Consistent with these findings, the transcriptional and translational regulation of apoptotic genes (BAX and Caspase-3) for cell apoptosis under HS was also influenced by the CAT gene. As shown in Figure 5C–E, 40 °C + NC significantly (*p* < 0.05) induced the expression of mRNA and protein levels of both BAX and Caspase-3 when compared with that in the NC group. Likewise, compared with both in the NC and 40 °C + NC groups, BAX and Caspase-3 showed a significant (*p* < 0.05) up-regulation in the 40 °C + CAT *si*RNA group both at the transcriptional and translational levels. Our findings show that the silencing of CAT promotes HS-induced apoptosis in GCs. Our findings depicted that CAT plays a regulatory role in the apoptosis of GCs.

### 3.7. Silencing of CAT Altered Cell Cycle Transition in GCs under HS

**Figure 6:**
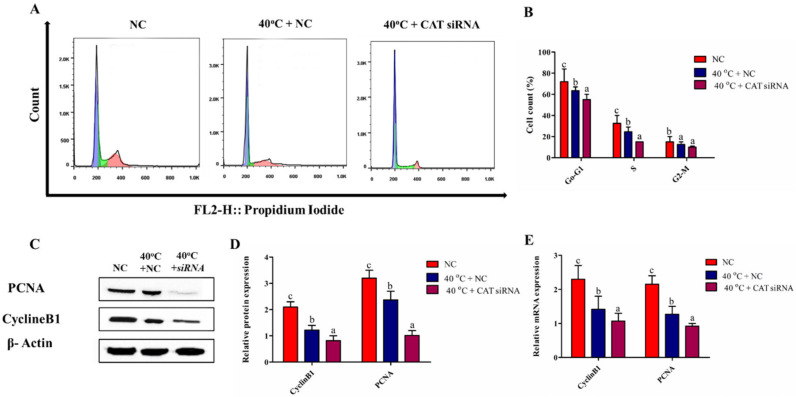
CAT regulates cell cycle distribution in GCs under HS. Flow cytometric profiles showed differences in cell cycle among treatment groups (**A**,**B**). The protein expressions of PCNA and CyclinB1 were analyzed by Western blotting (**C**,**D**). Moreover, the mRNA expression of PCNA and CyclinB1 from GCs of the NC and treated groups (40 °C + NC and 40 °C + CAT *si*RNA) were evaluated by RT-qPCR (**E**). Values are expressed as mean ± SEM of *n* = 3. Superscripts (a, b, c) represent a significant (*p* < 0.05) difference.

To check whether CAT regulates cell proliferation under HS, the cell cycle profile was assessed by nuclear DNA staining with PI using flow cytometry. The transition of the cell cycle phases was examined in the suggested groups (NC, 40 °C + NC, and 40 °C + CAT *si*RNA). Figure 6A,B show that after exposure of GCs to elevated heat, 40 °C + CAT *si*RNA resulted in a significant (*p* < 0.05) decrease in the percentage of cells in the G0/G1 phase with a subsequent decline in the S (DNA synthesis) phase and G2/M phase than that in NC. The above findings were further verified by the transcriptional and translational regulation of cell proliferation genes (PCNA and CyclinB1). PCNA and CyclineB1 mRNA and protein levels were significantly (*p* < 0.05) down-regulated in the 40 °C + NC and 40 °C + CAT *si*RNA groups than the NC group (Figure 6C–E). Taken together, the results clearly show that cells were arrested at the G0/G1 and G2/M phases to restore the HS-induced DNA damages, but the silencing of CAT further affected cell proliferation, depicting that CAT regulated cell cycle progression under HS.

### 3.8. Silencing of CAT Disrupted Mitochondrial Membrane Potential of GCs under HS

**Figure 7:**
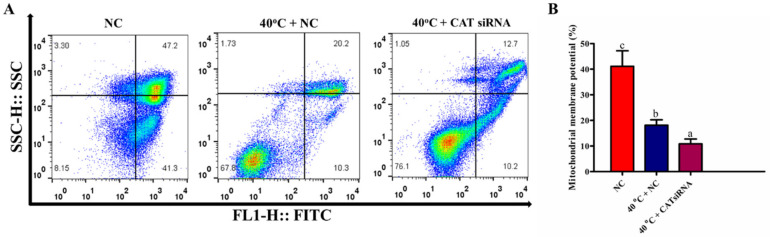
CAT regulates MMP of GCs under HS. Flow cytometric analysis for MMP of cultured GCs from the NC and treated (40 °C + NC and 40 °C + CAT *si*RNA) groups (**A**,**B**). Values are expressed as mean ± SEM of *n* = 3. Superscripts (a, b, c) represent a significant (*p* < 0.05) difference.

To examine whether the activity of CAT was effective in the regulation of the mitochondrial pathway, involved in GCs apoptosis under HS, the MMP of GCs was measured by FCM. Our findings revealed that compared with the NC group, MMP was significantly (*p* < 0.05) lower in the 40 °C + NC group. Similarly, MMP was significantly (*p* < 0.05) lower in the 40 °C + CAT *si*RNA group when compared with both the NC and 40 °C + NC groups (Figure 7A,B). Therefore, these results indicate that under HS, the CAT expression has a significant role in MMP regulation in GCs.

### 3.9. Silencing of CAT Impaired the Synthesis of P4 and E2 in GCs under HS

**Figure 8:**
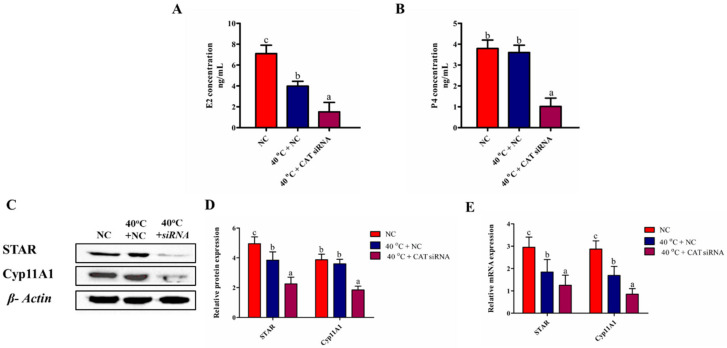
CAT regulates P4 and E2 synthesis in GCs under HS. Concentration of P4 and E2 in the culture medium released by the GCs in the different treated groups (40 °C + NC and 40 °C + CAT *si*RNA) and corresponding NC group (**A**,**B**). Western blot results of STAR and Cyp11A1 (**C**,**D**). The mRNA expression of STAR and Cy11A1 by RT-qPCR (**E**). Values are expressed as mean ± SEM of *n* = 3. Superscripts (a, b, c) represent a significant (*p* < 0.05) difference.

To evaluate the impact of CAT silencing on hormonal changes, we did ELISA for the estimation of the P4 and E2 concentrations. The results showed that the concentrations of P4 and E2 were significantly (*p* < 0.05) lower in the 40 °C + CAT *si*RNA group when compared with both in the NC and 40 °C + NC groups. Conversely, no significant change was observed for P4 between the NC and 40 °C + NC groups (Figure 8A,B). The above findings were further verified by the transcriptional and translational expression of steroidogenesis regulatory genes (STAR and Cyp11A1). STAR mRNA and protein levels were significantly (*p* < 0.05) down-regulated in the 40 °C + NC and 40 °C + CAT *si*RNA groups than those in the NC group. However, the protein expression of Cyp11A1 showed no significant difference between the NC and 40 °C + NC groups (Figure 8C–E). These results confirmed that the silencing of CAT under HS could decrease the concentration of P4 and E2 in GCs, and shows that CAT plays a vital role in the regulation of these hormones.

## 4. Discussion

**Figure 9:**
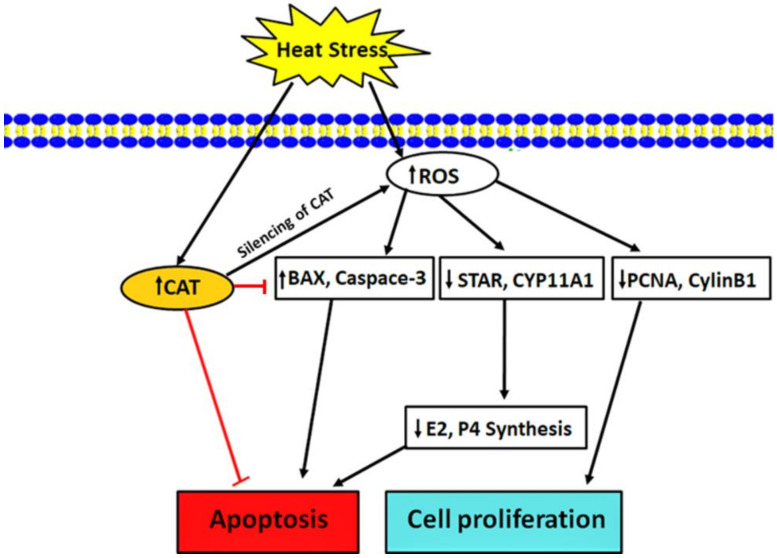
The mechanism of CAT regulating GCs apoptosis and cell proliferation under HS. Under HS, CAT prevents apoptosis by down-regulating the expression of BAX and Caspase-3, while the silencing of CAT induces ROS production and subsequently affects the level of E2 and P4 in GCs by regulating the STAR and Cyp11A1 genes, altering cell proliferation by down-regulating the PCNA and CyclinB1 genes.

An elevated ambient temperature is a major contributing factor to the low fertility of dairy cows during the summer season [40]. HS is a phenomenon that happens when an animal does not lose internal body heat sufficiently to sustain thermoregulation [41,42]. This HS is recognized to alter steroidogenesis [43,44], folliculogenesis [45,46], GCs function [47,48], oocyte maturation, fertilization, and embryonic development [49,50], thereby resulting in an impaired reproductive efficiency in animals. In the current study, we revealed a key role of CAT in regulating the apoptosis and proliferation of GCs under HS. In general, our findings verified that HS can up-regulate the CAT expression, while in particular, we found that CAT can significantly regulate intracellular ROS production, apoptosis, cell proliferation, MMP, and hormone synthesis in GCs. Figure 9 illustrates a brief description of the CAT gene regulating GC functions under HS.

In the current study, we report the successful in vitro knockdown of CAT in bovine GCs. We validated the knockdown efficacy of *si*RNA in GCs and found the down-regulation of mRNA and protein levels of CAT. After successful knockdown, GCs were exposed to different treatment groups (40 °C + NC, 40 °C + CAT *si*RNA) and corresponding NC group. It is well-known that HS is responsible for inducing ROS production, resulting in oxidative stress [51,52] and cell apoptosis [53], which afterwards compromise fertility [54,55]. In our previous work, we confirmed that HS at 40 °C significantly increased intracellular ROS accumulation and apoptosis in GCs [52]. Some other studies found that pro-apoptotic genes (BAX and Caspase 3) were also up-regulated under HS, through the apoptosis signaling pathway [21,56,57]. The MMP disruption due to the up-regulation of apoptotic genes could result in apoptosis [58]. CAT has long been postulated as a main line of defense against ROS-mediated cellular damage by scavenging H_2_O_2_. Furthermore, CAT is also responsible for the breakdown of H_2_O_2_ into O_2_ and H_2_O, thus protecting cells from oxidative damage [30]. Our findings suggested that CAT silencing increased the intracellular ROS abundance, thereby having a key role in the suppression of apoptosis in GCs. The apoptotic factors were further validated by the quantification of both mRNA and the protein expression of BAX and Caspase-3 in these cells. We found an increase in the expression of BAX and Caspase-3 both at the transcriptional and translational levels in the 40 °C + CAT *si*RNA group. This indicated that *CAT* silencing results in oxidative stress that promoted mitochondrial-mediated apoptosis in GCs.

A high ambient temperature affects fertility by altering ovarian cells, the cell cycle, MMP, and inducing apoptosis [55,59,60,61,62]. The HS-mediated disruption of MMP is the main reason resulting in cell apoptosis, and damage to MMP is one of the indicators of cell death [63]. Likewise, the release of cytochrome c from mitochondria into the cytosol is also an indication for mitochondrial damage. The activation of Caspase-3 under the influence of cytochrome c results in DNA fragmentation and condensation of chromatin [30,64]. Furthermore, in swine GCs, PCNA, CyclinB1, Caspase-3, and BAX are considered as important regulators for the proliferation and apoptotic pathway [65,66]. PCNA has long been known as a cell proliferation indicator and its expression mainly occurs during the S phase and several other proteins including CDK2 and the CDK inhibitor p21 affect the propagation of the cell cycle through interactions with PCNA [67]. Additionally, the expression of CyclinB1 mainly occurs during the G2/M phase of the cell cycle, thereby its regulation is required for the transition of G2 to the M phase [68]. Swine GCs cultured under HS conditions in vitro down-regulated the expression of PCNA and cyclinB1 [60]. Some cell cycle regulators could influence both cell proliferation and cell apoptosis. One of the concerns of the current study is to establish the association between apoptosis, altered MMP, and cell cycle arrest due to the silencing of the CAT gene in GCs under HS, which has not been previously reported. Our results revealed that the silencing of CAT led to a significant decrease in MMP and arrest in the G1 cell cycle phase. CAT silencing also diminished the propagation of GCs and decreased the cell count in the G1 and the S phases. We measured the differential mRNA and protein regulation of PCNA and CyclinB1. Our findings indicated that the silencing of CAT significantly down-regulated these genes. This indicated that CAT had a very crucial role in controlling the MMP and cellular progression in GCs under HS.

In addition, HS has influenced the regulation of genes, i.e., STAR and Cyp11A1, associated with steroidogenesis (E2 and P4) [52]. It was suggested that E2 biosynthesis in the ovarian follicles is promoted by the positive regulation of Cyp11A1 [69]. Moreover, P4 is a key steroid hormone for the regulation of bovine cyclicity, and its secretion is governed by the regulation of STAR and Cyp11A1 [70,71,72]. Furthermore, it has been previously found that P4 concentrations were not different between the control and HS groups under HS even under the down-regulation of STAR and Cyp11A1 [73]. Some other researches recorded that HS results in an increased biosynthesis of steroid hormones in swine GCs [74]. In addition, evidence from a previous investigation showed that E2 can dampen GCs’ apoptosis under HS [75]. Our results clearly demonstrate that HS can impair the E2 levels in GCs, and the regulation of CAT has an influence on cell apoptosis by affecting the E2 and P4 levels. We found that the silencing of CAT further decreased the concentration of E2 and P4 by down-regulating the expression of the STAR and Cyp11A1 genes both at the transcriptional and translational levels. Therefore, based on our findings, we can postulate that the regulation of the CAT gene under HS can regulate apoptosis by impacting the synthesis of E2 and P4 in GCs under HS [75]. Our work can be extended further to understand the mechanism of oocyte modulation and embryo development in response to HS under the regulation of CAT.

## 5. Conclusions

In this study, we are the first to explore in detail the mechanism of CAT regulating apoptosis and cell cycle progression in bovine GCs under HS. Using an in vitro model, our results disclose that the silencing of CAT under HS is involved in compromising the physiological functions of GCs by increasing the intracellular accumulation of ROS, induction of apoptosis, disruption of MMP, impaired E2 and P4 production, and altered shift in the cell cycle transition. This was further proved by the associative apoptotic, steroidogenic, and cell cycle genes (BAX, Caspase-3, STAR, Cyp11A1, PCNA, and CyclinB1). Our findings propose a baseline study for dairy cattle selection under hot climates.

## Figures and Tables

**Table 1:** List of gene primers used for RT-qPCR.

Gene	Accession No.	Forward 5′→3′	Reverse 5′→3′
CAT	NM_001035386.2	GTTCGCTTCTCCACTGTTGC	AGGTGCGTTTGAGGGTTTCT
BAX	XM_003355974.2	GGCTGGACATTGGACTTCCTTC	TGGTCACTGTCTGCCATGTGG
Caspase-3	XM_005671704.1	CTGGACTGTGGCATTGAGAC	GCAAAGGGACTGGAGAACC
CyclinB1	NM_001170768.1	AAGACGGAGCGGATCCAAAC	CCAGTGACTTCACGACCCAT
PCNA	NM_001291925.1	GCGTTCATAGTCGTGTTCCG	TTCAAGATGGAGCCCTGGAC
STAR	NM_174189.3	CCCATGGAGAGGCTTTATGA	TGATGACCGTGTCTTTTCCA
Cyp11A1	NM_176644.2	CTGGCATCTCCACAAAGACC	GTTCTCGATGTGGCGAAAGT
GAPDH	NM_001034034.2	GGTGCTGAGTATGTGGTGGA	GGCATTGCTGACAATCTTGA
